# Examining the utility of a laser device for measuring height in free-living adults and children

**DOI:** 10.1186/s12937-015-0082-4

**Published:** 2015-09-08

**Authors:** Sandra N. Mayol-Kreiser, Vanessa M. Garcia-Turner, Carol S. Johnston

**Affiliations:** School of Nutrition and Health Promotion, 500 North 3rd Street, Phoenix, AZ 85004 USA

## Abstract

**Background:**

Height is an important health assessment measure with many applications. In the medical practice and in research settings, height is typically measured with a stadiometer. Although lasers are commonly used by health professionals for measurement including facial imaging, corneal thickness, and limb length, it has not been utilized for measuring height. The purpose of this feasibility study was to examine the ease and accuracy of a laser device for measuring height in children and adults.

**Findings:**

In immediate succession, participant height was measured in triplicate using a stadiometer followed by the laser device. Measurement error for the laser device was significantly higher than that for the stadiometer (0.35 and 0.20 cm respectively). However, the measurement techniques were highly correlated (*r*^2^ = 0.998 and 0.990 for the younger [<12 y, *n* = 25] and older [≥12 y, *n* = 100] participants respectively), and the estimated reliability between measurement techniques was 0.999 (ICC; 95 % CI: 0.998,1.000) and 0.995 (ICC; 95 % CI: 0.993,0.997) for the younger and older groups respectively. The average differences between the two styles of measurement (e.g., stadiometer minus laser) were significantly different from zero: +0.93 and +0.45 cm for the younger and older groups respectively.

**Conclusions:**

These data demonstrate that laser technology can be adapted to measure height in children and adults. Although refinement is needed, the laser device for measuring height merits further development.

## Introduction

Height is used to calculate body mass index, the most commonly applied metric for placing individuals and populations in weight categories [1, 2]. Height is also used to assess growth and nutritional adequacy in children [3–5], and it is used in predictive equations to estimate other health parameters including metabolic rate and lean body mass [6, 7]. In research settings, height is typically measured with a stadiometer, a portable device composed of a vertical backboard and adjustable head piece, with a reported measurement error of 0.2–0.3 cm [8].

Lasers are commonly used in the medical field for measurement including facial imaging, corneal thickness, and limb length. In these applications, lasers are easy to operate and possess high measurement reliability [9, 10]. Although, lasers are not used in the medical field to measure height, veterinarians use laser devices to measure animal height. In comparison to a conventional measuring stick, a laser device for measuring height at the withers of horses and ponies was demonstrated to be reliable and accurate [11]. The purpose of this study was to examine the feasibility of a laser device for measuring height in children and adults and to compare these values with those recorded using a stadiometer.

## Methods and procedures

### Participants and recruitment

One hundred and twenty eight individuals (3–80 y) participated in the study and completed all measurements. The study was approved by the Institutional Review Board at Arizona State University, and written consent, parental consent, and child assent were collected as appropriate.

### Laser device

**Fig. 1:**
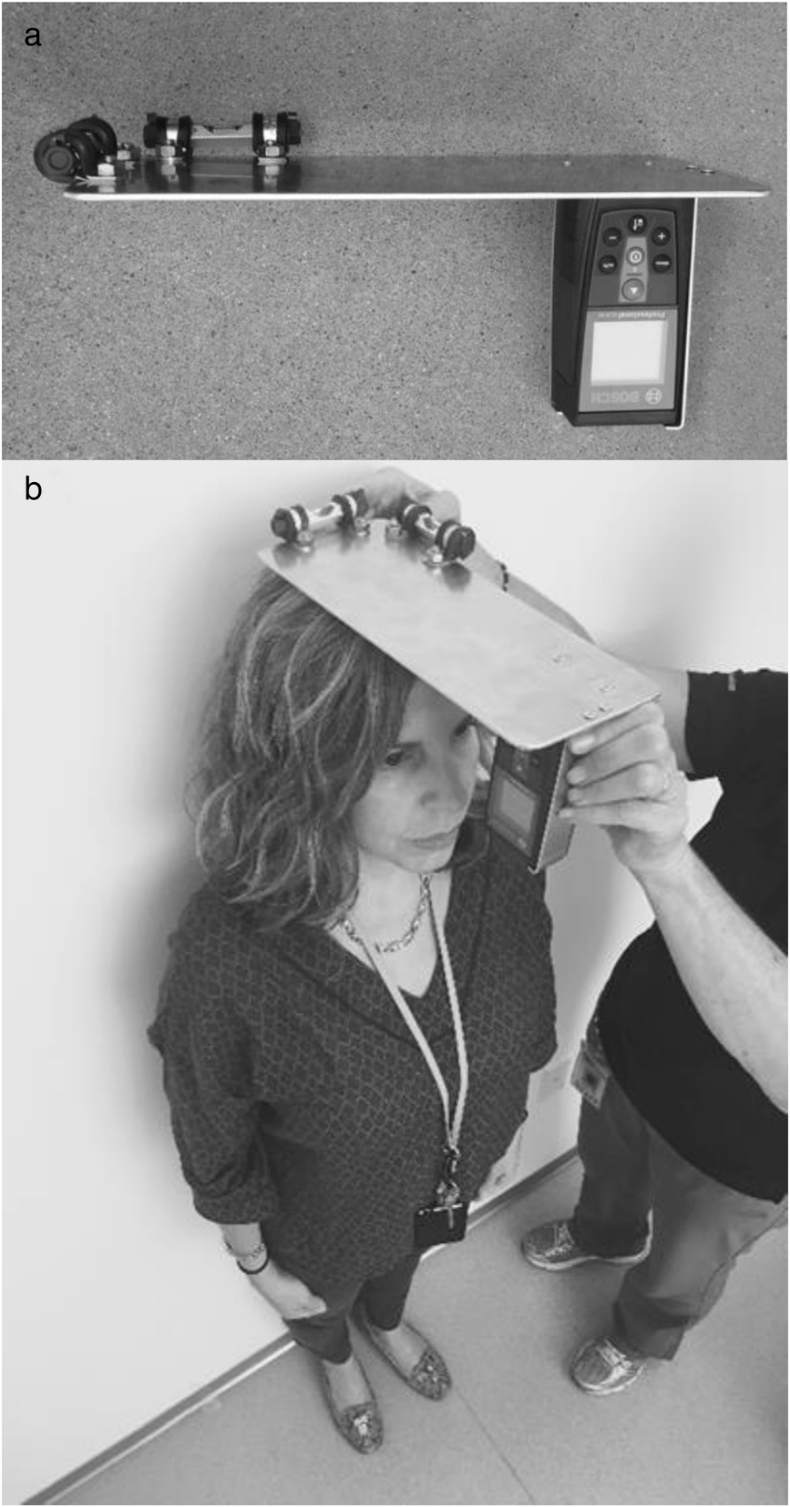
**a** Laser device. **b** The laser device is shown positioned on the top of the skull. Once the device is leveled on the horizontal plans, the laser is activated, and a digital display indicates the distance in feet plus inches to the nearest thousandth

The laser device weighed 527 g (Fig. 1). Two line levels were attached to adjacent edges of a metal plate (11 x 27.7 cm) using adel clamps to enable the user to position the metal plate parallel to the ground. A compartment for the laser (Bosch GLM40, Robert Bosch Tool Corp., Mt. Prospect, IL) was attached perpendicular to the metal plate, and when positioned on the top of the skull, the metal plate represented the y-axis and the laser beam was the z-axis. This positioning pointed the laser to the ground in front of the subject permitting the user to measure the distance from the top of the skull to the ground.

### Study procedure

A single experimenter completed all height measurements on a hard, flat surface, and for each measurement, shoes were removed, heals were positioned against a wall [or the back of the stadiometer], and the head was positioned with the Frankfurt plane parallel to the ground. Seventy-seven percent of participants were measured outdoors. Three consecutive measurements were made using a mobile stadiometer (Seca 213 Portable Measuring Rod, Seca Corporation, Hanover, MD). The participant stepped off the stadiometer between each measurement, and height was manually recorded in centimeters to the nearest tenth. The laser measurements were conducted immediately following the stadiometer measurements at an adjacent location against a wall. The laser device was rested on the top of the skull, and once leveled, the laser was activated. A digital display indicated the distance in feet plus inches to the nearest thousandth. This procedure was repeated to provide three separate measures. The laser dot was visualized at the participants’ feet before each measurement was taken. Laser height measures were converted to centimeters prior to analyses.

### Statistical analyses

Triplicate measurements were averaged to provide a single height measurement per participant per technique, and data are reported as the mean ± SD. Three outliers for the laser measurement were identified (>3 SD from the mean) and removed prior to analyses. Error in the height measurements by technique were expressed as the SD for the triplicate measures. Differences between measurement styles were calculated by subtracting the mean laser measure from the mean stadiometer measure. Univariate Analysis of Variance was used to assess differences between means, and a one sample *t*-test was employed to assess whether means differed from zero. To compare the measurement devices, participant data were grouped by age (<12 and ≥ 12 y; *n* = 25 and 100 respectively), and mean heights were normally distributed within groups. Intraclass correlation (ICC) analyses for reliability measurement between devices and Bland and Altman plots (differences between measurement means plotted against the means) were used to assess measurement reliability between the laser device and stadiometer. Analyses were performed using SPSS software, version 22 (2013, IBM-SPSS Inc). A *P* value <0.05 was considered significant.

## Results

There were 9 males and 16 females in the younger group (7.8 ± 2.7 y) and 36 males and 64 females in the older group (31.6 ± 17.6 y). Heights (measured via stadiometer) averaged 143.9 ± 13.3 and 125.0 ± 20.5 cm (*p* = 0.010) and 173.3 ± 12.0 and 165.7 ± 7.0 cm (*p* = 0.001) for the males and females in these groups respectively. The triplicate measurements for both devices were highly reliable (ICC [95 % CI] = 1.00 [1.00,1.00] and 0.991 [0.988,0.994] for the stadiometer and laser device respectively). Measurement error was greater for the laser device as compared to the stadiometer (SD = 0.35 and 0.20 cm for the laser device and stadiometer respectively, *P* < 0.001). Measurement error by technique did not differ by age group or gender; however, measurement error was significantly higher for the laser technique when the measurement was taken outdoors as compared to indoors (0.39 versus 0.21 cm respectively). Measurement error was not impacted by location for the stadiometer technique (0.20 and 0.19 cm for outdoors and indoors respectively).

**Fig. 2:**
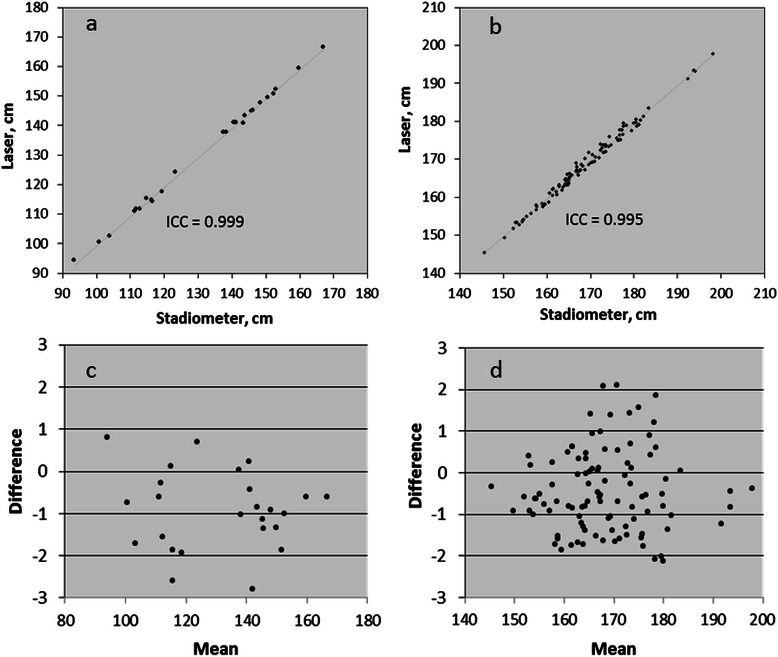
Scatterplots for the laser and stadiometer measurements in individuals (**a**) <12 y [*n* = 25] and (**b**) ≥ 12 y [*n* = 100]; and Bland and Altman plots for the laser and stadiometer measurements in individuals (**c**) <12 y [*n* = 25] and (**d**) ≥ 12 y [*n* = 100]

Examination of the mean heights for the younger and older groups based on measurement technique suggests that the laser device measured height slightly below that of the stadiometer method (Table 1). The average differences between the two styles of measurement (e.g., stadiometer minus laser) were significantly different from zero: +0.93 ± 0.92 and +0.45 ± 0.98 cm for the younger and older groups respectively (Table 1). Since, in practice, height is not measured in triplicate, average differences were also computed for the first measurements (+0.90 ± 1.05 and +0.29 ± 1.08 cm for the younger and older groups respectively; both values differing significantly from zero). Differences between measurement styles were not impacted by gender or location in either age group. The estimated reliability between measurement techniques was 0.999 (ICC; 95 % CI: 0.998,1.000) and 0.995 (ICC; 95 % CI: 0.993,0.997) for the younger and older groups respectively (Table 1; Fig. 2(a) and (b)). The measurement techniques were highly correlated (*r*^2^ = 0.998 and 0.988 for the younger and older groups respectively). The Bland-Altman plots for the data by age group are displayed in Fig. 2(c) and (d).

**Table 1:** Measurement of height using a laser device or stadiometer in individuals <12 or ≥12 y of age^a^

	<12 y	≥12 y
*n*	25	100
Stadiometer, cm	131.8 ± 20.2	168.5 ± 9.7
Laser device, cm	130.9 ± 20.1	168.0 ± 9.8
*P* value^b^	<0.001	<0.001
Average difference,^c^ cm	0.93 ± 0.92	0.45 ± 0.98
95% limits of agreement	−0.88,2.74	−1.48,2.37
Intraclass correlation^d^	0.999	0.995
95 % Confidence interval	0.998,1.000	0.993,0.997

^a^Values are the mean ± SD and represent the average of three consecutive measurements. Three laser measurement outliers (>3 SD from the mean) were removed

^b^Univariate analysis of variance

^c^Values are the mean ± SD for the difference between the stadiometer measurement and the laser device measurement; 95 % limits of agreement = (average difference) ±1.96(SD of difference)

^d^Intraclass correlation for reliability between device measurements

## Discussion

Time-of-day, posture, and inspiration status significantly impact height [8, 12, 13], but improper installation of measuring instruments may be the most common source of error in height measurement [8]. Laser devices are commonly used commercially to accurately measure heights and distances quickly and with considerable ease; however, the use of lasers to measure height in healthcare and research settings has not been investigated. Heights measured using the laser device and a stadiometer were highly correlated in children and in adults. Moreover, the laser device demonstrated excellent measurement reliability. However, measurement error for the laser device was significantly higher than that for the stadiometer (0.35 and 0.20 cm respectively), and although these values differ significantly, they closely mirror the error range previously reported for height measurement using stadiometers (0.2–0.3 cm) [8]. The hand-held laser device developed for this research required adjustment of two levels simultaneously, a possible source of measurement error. Furthermore, use of the laser device outdoors increased error. Future devices mounting the laser in a level, fixed manner would simplify its use and likely reduce error.

The laser device measured height slightly below that recorded by the stadiometer (−0.5 cm for individuals 12 y and older). Others also reported (in horses) that the laser device measured height below that recorded using a conventional measuring stick (−0.3 cm) [11]. Hence, there appears to be a bias for laser devices to measure height below that recorded using a stadiometer. This bias is also evident in the Bland and Altman plots for the younger and older age groups (Fig. 1(c) and (d)), yet other systematic biases (such as increased error as height increased) were not apparent suggesting that the laser device accuracy may improve with refinement. To promote accuracy, the laser device utilized two levels to assure a parallel plane to the ground. However, the skill needed to adjust the levels correctly prior to activating the laser beam may reduce accuracy. Also, since the laser device rests directly on the skull, it may compress the hair and skin to a greater degree than the stadiometer headpiece accounting for the shorter measurement. The difference in height measurement between the laser device and stadiometer was higher for the younger versus older age group. Children have difficulty understanding instructions and maintaining correct posture during height measurement [8]. The use of a laser likely contributed to this variation since many of the children wanted to look down to see the laser dot when the laser was activated. Standardized protocols for the measuring process in children will help to reduce this error.

Laser technology has advanced the ease and accuracy of measurement in many professions, but this technology has not been examined for measuring height in clinical or research settings. Utilizing a laser system to measure height would not reduce the common sources of error in measuring height (posture, time-of-day, etc.); however, once perfected, laser measurement would be rapid and versatile. In clinical settings and research units, the laser could be mounted for ease of use, and a hand held version could be developed for field use. Moreover, a laser device could be used to measure individuals in a supine position as well as the standing position, a feature not available with stadiometers.

## Conclusion

A laser device for measuring height merits further development. Individual stature is used in many applications including assessing growth adequacy and placing individuals into weight categories. Utilizing laser technology for height assessment has the potential to improve accuracy and ease of measurement.
